# Analysis of the REJ Module of Polycystin-1 Using Molecular Modeling and Force-Spectroscopy Techniques

**DOI:** 10.1155/2013/525231

**Published:** 2013-05-26

**Authors:** Meixiang Xu, Liang Ma, Paul J. Bujalowski, Feng Qian, R. Bryan Sutton, Andres F. Oberhauser

**Affiliations:** ^1^Department of Neuroscience and Cell Biology, University of Texas Medical Branch, Galveston, TX 77555, USA; ^2^Department of Biochemistry and Molecular Biology, University of Texas Medical Branch, Galveston, TX 77555, USA; ^3^Department of Medicine, Division of Nephrology, University of Maryland School of Medicine, Baltimore, MD 21201, USA; ^4^Department of Cell Physiology and Molecular Biophysics, Texas Tech University Health Sciences Center, Lubbock, TX 79430, USA; ^5^Sealy Center for Structural Biology and Molecular Biophysics, University of Texas Medical Branch, Galveston, TX 77555, USA

## Abstract

Polycystin-1 is a large transmembrane protein, which, when mutated, causes autosomal dominant polycystic kidney disease, one of the most common life-threatening genetic diseases that is a leading cause of kidney failure. The REJ (receptor for egg lelly) module is a major component of PC1 ectodomain that extends to about 1000 amino acids. Many missense disease-causing mutations map to this module; however, very little is known about the structure or function of this region. We used a combination of homology molecular modeling, protein engineering, steered molecular dynamics (SMD) simulations, and single-molecule force spectroscopy (SMFS) to analyze the conformation and mechanical stability of the first ~420 amino acids of REJ. Homology molecular modeling analysis revealed that this region may contain structural elements that have an FNIII-like structure, which we named REJd1, REJd2, REJd3, and REJd4. We found that REJd1 has a higher mechanical stability than REJd2 (~190 pN and 60 pN, resp.). Our data suggest that the putative domains REJd3 and REJd4 likely do not form mechanically stable folds. Our experimental approach opens a new way to systematically study the effects of disease-causing mutations on the structure and mechanical properties of the REJ module of PC1.

## 1. Introduction

**Figure 1:**
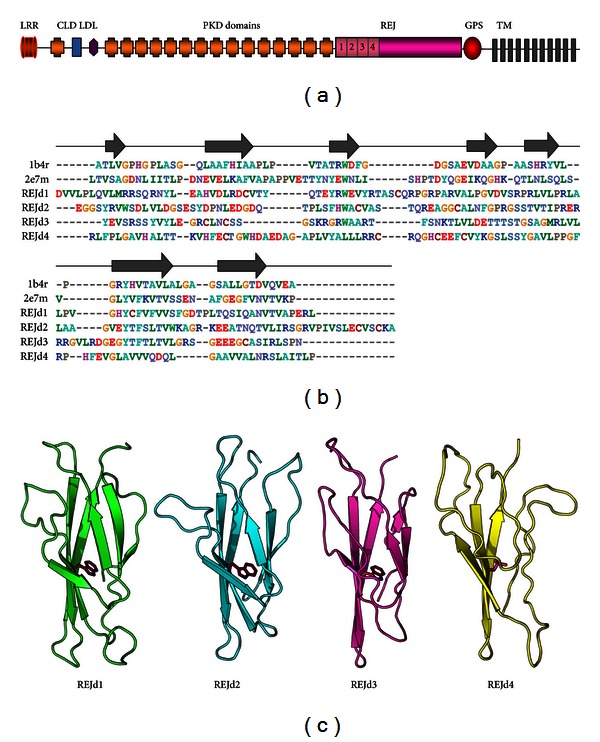
(a) Diagram of the predicted domain architecture of the extracellular region of PC1. The ectodomain has a large collection of domains: several leucine-rich repeats (LRR), a C-type lectin domain ((CLD) blue box), an low-density-lipoprotein-like domain ((LDL-A domain) purple octagon), 16 PKD domains (boxes in orange), and the 1000 aa long Receptor for Egg Jelly (REJ), in purple) region. GPS: G-protein coupled proteolytic site. TM: transmembrane domains. (b) Sequence alignments of the putative REJ domains with template structures of the human PKD domain no. 1 from polycystin-1 (1b4r) and the human PKD domain from protein KIAA0319 (2e7m). The arrows indicate beta-stranded secondary structure regions and are derived from the predicted secondary structure of 1b4r as calculated by the DSS algorithm in PyMol. The predicted secondary structure for 2e7m shares similar characteristics. The color of the various amino acids in the alignment reflects the chemical composition of the residues in the REJ fold, for example, red = acidic, blue = basic, and green = hydrophobic. (c) Homology models of putative FNIII domains within the REJ module of human PC1. The conserved Trp residue in REJd1, -d2, and -d3 is shown in purple.

PC1 is a large transmembrane protein, which, when mutated, causes autosomal dominant polycystic kidney disease (ADPKD), one of the most common life-threatening genetic diseases that is a leading cause of kidney failure [1]. PC1 may have a role in sensing of flow [2, 3], pressure [4] and the regulation of the cell cycle [5] and cell polarity [6]. PC1 may sense signals from the primary cilia, neighboring cells, and extracellular matrix and transduces them into cellular responses that regulate proliferation, adhesion, and differentiation that are essential for the control of renal tubules and kidney morphogenesis [1, 3, 7, 8]. The predicted amino acid sequence of PC1 (Figure 1(a)) suggests that it is a large multidomain membrane protein with 11 transmembrane domains. Its N-terminal extracellular region contains 4 leucine-rich repeats ((LRR) 250 amino acid long), a C-type lectin domain ((CLD) 130 amino acid long), a low-density-lipoprotein-like domain (LDL-A domain), 16 Ig-like domains (PKD domains, each 90 amino acid) and a region that is homologous to a sea urchin protein called receptor for egg jelly (REJ) [9, 10]. The PKD domains in PC1 have a similar topology fibronectin type III (FNIII) domain found in other modular proteins with structural and mechanical roles (recently reviewed in [11]). PC1 interacts with polycystin-2 (PC2) in the primary cilia of renal epithelial cells which forms a mechanically sensitive ion channel complex. Bending of the cilia induces Ca^2+^ flow into the cells, mediated by the PC1-PC2 complex [2, 3, 12]. Mechanical signals are thus transduced into cellular responses that regulate proliferation, adhesion, and differentiation, essential for the control of renal tubules and kidney morphogenesis. Using SMFS, we and others have shown that the PC1 N-terminal extracellular region is highly extensible and that this extensibility is mainly caused by the unfolding and refolding of its PKD domains [13–15]. These force-driven reactions are likely to be important for cell elasticity and the regulation of cell signaling events mediated by PC1.

 The REJ module is a major component of PC1 ectodomain that extends to about 1000 amino acids. A large number of mutations map on to this region. According to the Mayo Clinic PKD database, there are about 230 mutations including 80 missense mutations in the REJ region and of those about 65 missense mutations are predicted to be disease-causing mutations, highlighting the importance of this region for PC1 function. However, very little is known about the structure or function of this module. Recent evidence shows that PC1's ectodomain undergoes cleavage at the G-protein coupled proteolytic site (GPS), a process that requires the complete REJ region [16–18]. GPS cleavage is a process that is essential for kidney structure and function, as shown by the Pkd1^V/V^ knock-in mouse [19], as well as by the fact that a number of mutations in the REJ indeed disrupt GPS cleavage [20, 21].

 The REJ of PC1 shares similarity to the sea urchin sperm REJ proteins (such as SpREJ1, SpREJ2, and SpREJ3) and other members of the PC1 family (such as PKDREJ and PKD1L1) [22]. Initial secondary structure analysis predicted a total of four FNIII repeats in the first 400 amino acids of the REJ module of PC1 [23]. A later work concluded that the PC1 REJ module represents a novel sequence that contains no repeating motifs, and it does not show any homology to any known fold [9]. However, subsequent SMFS experiments indicated the existence of FNIII type of domains within the REJ module [14]. 

 More recently, Schröder et al. used comprehensive sequence analysis together with CD spectroscopy and NMR techniques to analyze the first 425 amino acids of the REJ module [24]. They found that within this segment there are total of four predicted FNIII domain but only the first two domains could be expressed as soluble proteins, and only domain 2 was amenable for NMR analysis. Their data show that domain 2 has all the features of a bona-fide FNIII domain. The biophysical analysis of domain 1 was hindered because of partial aggregation. Domain 3 expressed well but in inclusion bodies and degraded quickly. Domain 4 expressed extremely poorly and in inclusion bodies.

 In this work we used a different approach, where we combined homology modeling, protein engineering, and SMFS to systematically characterize the stability of the predicted four FNIII domains in the first 425 amino acids of the REJ module. After flanking the different putative FNIII sequences with titin I27 or MBP domains, we found that these constructs express well as soluble proteins in *E. coli* and were able to analyze their mechanical stability. We demonstrate that the REJ module contains several stable domains that are likely to have a fold similar to FNIII domains, confirming our previous predictions [14]. Our approach should make the analysis of the biophysical effects of mutations on the REJ module possible.

## 2. Materials and Methods

### 2.1. Homology Modeling

 Multiple sequence alignment was performed with ClustalW (version 2.1, [25]) and visualized with JalView. We chose the best model based on the lowest calculated model energy values (molpdf) as reported by MODELLER (version 9.9, [26]) and low DOPE scores for each model [27]. Structures were rendered using PyMol (http://www.pymol.org/). In the homology modeling analysis of the putative REJ domains we used template structures of the human PKD domains from polycystin-1 (1b4r) and from protein KIAA0319 (2e7m). Our assumption was that the REJ domains were a continuation of the PKD repeats from the more N-terminal domains. Since 1B4R and 2e7m shared at least some sequence similarity with the REJ regions, we picked these templates. We initially attempted to use both 1b4r and 2e7m to model all four REJ domains to strengthen the quality of the final model. However, due to the sequence degeneracy and the low overall sequence identity in each of the four sequences, the quality of the resulting homology models was poor as judged by the DOPE scores. We assumed that both the Trp residue in beta-strand B and the Tyr residue in beta-strand E make up essential elements as core residues. REJd1 and REJd2 could be most optimally aligned with 1b4r, based on local sequence homology with conserved secondary structure elements, while REJd3 and REJd4 could be aligned with 2e7m. The initial alignments against each of target structures (1b4r and 2e7m) were performed with Clustal; however, the alignments that were used for the homology models were manually adjusted to optimize the chemical nature of more conserved amino acids in each domain. Further, the perresidue DOPE analysis of REJd1 and REJd2 correlated best with using 1b4r as a template structure, while the perresidue DOPE analysis of REJd3 and REJd4 correlated best with 2e7m.

### 2.2. Construction, Expression, and Purification of REJ Segments for SMFS and CD Experiments

In order to characterize the mechanical properties of putative FNIII domains in the REJ module we made several protein constructs (Table 1) and expressed these in either *E. coli* or insect cells. Recombinant DNA techniques and multiple step cloning technique were used to construct different REJ segments heteropolyproteins [13, 14, 28, 29]. For *E. coli *expression system, REJ constructs were introduced into a modified pRSET A vector [28] or a p202 vector and expressed in *E. coli* BL21 or C41 strains. The p202 vector contains a maltose-binding protein (MBP) sequence upstream of the multicloning site to increase the solubility of the target protein. The proteins were purified by Ni-affinity chromatography as previously described [13–15, 30]. The proteins were kept in PBS containing 5 mM DTT (in order to prevent dimer formation since the I27 constructs have cysteine residues at the C-terminus to facilitate attachment to the gold coated AFM tip). For the insect expression system, REJ constructs were introduced into pVL1392 vector or pFastBac vector and expressed in insect cell Sf9, using the BaculoGold Transfection kit (BD Biosciences). All the constructs were cotransfected via Baculovirus Expression Vector System (BD Biosciences) into host cell Sf9 and cultured in Insect-Xpress w/L-Gln medium (LONZA Walkersville, Inc.) supplied with 5–10% FBS and penicillin/streptomycin. The infection and amplification protocols were based on the manual of BD BaculoGold Transfection kit. After 3 rounds of amplification of the recombinant baculoviruses, the cell pellets and supernatant were collected. The cell pellets were lyzed in insect cell lysis buffer (BD Biosciences) supplied with protease inhibitors (Roche) on ice bath for 30 min and sonicated. The proteins were purified in native conditions with Ni-NTA resins and stored at 4°C for AFM studies. 

 We found that the REJd1-4, REJd1, and REJd4 recombinant constructs are expressed poorly as insoluble proteins in insect cells and bacteria. This is in agreement with a recent study that found that REJd1, -d2, -d3, and -d4 are very hard to express in *E. coli* [24]. In order to increase their solubility we flanked the REJ domains with maltose-binding protein (MBP) and titin I27 domains. We found that the MBP-REJd1-I27 and MBP-REJd1,2-I27 constructs are expressed as soluble proteins in *E. coli*. However, the MBP-REJd3,4-I27 is expressed poorly and mostly in inclusion bodies even at low induction temperatures (16°C). To further increase the solubility we flanked the REJd4 and REJd3,4 sequence with multiple titin I27 domains, (I27)_3_-REJd4-(I27)_2_ and (I27)_3_-REJd3,4-(I27)_2_. These constructs are expressed well as soluble proteins in both bacteria and insect cells. Our original plan was to characterize the secondary structure and thermodynamic stability of the different REJ proteins using far-UV CD and Equilibrium Denaturation techniques. However, we were unable to accomplish this goal for the following reasons: (i) we found that the native REJd1-4, REJd1, and REJd4 recombinant proteins are expressed poorly as insoluble proteins in both insect cells and in bacteria; (ii) in the MBP-REJd1-I27 and the MBP-REJd1,2-I27 constructs we included protease cleavage sites (TEV and thrombin) in between the MBP and REJd1 sequences. We found that, after cleavage, both proteins precipitated as an insoluble product; (iii) we were unable to make the (I27)_3_-REJd1-(I27)_2_ or (I27)_3_-REJd2-(I27)_2_ constructs; (iv) the only protein that expressed well enough for CD analysis was the (I27)_3_-REJd4-(I27)_2_ construct.

### 2.3. Single-Molecule Force Spectroscopy

The mechanical properties of single proteins were studied using a home-built single-molecule atomic force microscope (AFM) as previously described in [31]. The spring constant of each individual cantilever (MLCT or Olympus OBL, Veeco Metrology Group) was calculated using the equipartition theorem [32]. In a typical experiment, a small aliquot of the purified proteins (~1–10 *μ*L, 10–100 *μ*g/mL) was allowed to adsorb onto a Ni-NTA coated glass coverslip [33, 34] for about 5 min and then rinsed with PBS. The pulling speed was in the range of 0.5–0.7 nm/ms. In single-molecule force-spectroscopy experiments the probability of picking up a protein is characteristically very low because the density of molecules has to be low enough to pull single molecules. Hence, in about 95% of the experiments, the approach of the AFM tip to the surface does not result in a contact with a protein [35, 36]. In addition the protein is contacted at random locations by the AFM tip and most does not show complete unfolding of the REJ protein construct. The AFM recordings traces were selected using the following criteria: (i) the trace should have clean initial force extension after retraction from the surface (i.e., little or no unspecific interactions); (ii) traces should have detachment forces higher than 200 pN to be sure that the protein is completely extended and unfolded. We chose the 200 pN threshold because most studied protein domains unfold at forces less than this force [37]. We found that typically about 1 in 500–1000 of force-extension traces fulfilled these criteria.

### 2.4. Contour Length Measurements

The initial contour length of the folded protein (Lc) and the contour length increments (ΔLc) caused by domain unfolding were measured using the worm like chain (WLC) equation. The adjustable parameters of the WLC model are the persistence length, *p* and the contour length of the polymer [38, 39]. We measured Lc by manually fitting the first force peak of the sawtooth pattern to the WLC equation; the zero length point was defined as the point where the AFM cantilever tip contacts the coverslip. In a typical experiment, the cantilever tip is pressed into a layer of purified protein adsorbed onto a glass coverslip. Protein molecules are then stretched. Experimentally we find that the proximal region of the force-extension recording is frequently contaminated with nonspecific interactions due to entanglement with other protein molecules, making it difficult to get a clean estimation of zero-force-zero-length point. These nonspecific interactions can account to about 10–30 nm of the initial stretching region.

### 2.5. Circular Dichroism

 The far UV CD spectra of the titin I27 and the I27_3_-REJd4-I27_2_ polyprotein were recorded on a Jasco J-815 Spectropolarimeter. A 0.2 cm path length cuvette was used as the sample container. The protein concentration was 1 *μ*M in 10 mM phosphate buffer. The data reported in Figure S1 corresponds to the average of 3 scans obtained at a scan rate of 50 nm/min in the range of 200–260 nm (see Suplemetary Material available online at http://dx.doi.org/10.1155/2013/525231). The secondary structure content was estimated using the CDNN program (version 2.0.3.188) [40].

### 2.6. Steered Molecular Dynamic (SMD) Simulations

 We simulated the force-induced, linear unfolding of each putative REJ domain using Steered Molecular Dynamics as implemented in the GPU-accelerated version of NAMD [41, 42]. Coulombic forces were restricted using the switching function from 10 Å to a cutoff at 12 Å. The CHARMM22 force field was used throughout the simulations. Each of the REJ domain models was solvated in a water sphere with a boundary of 15 Å. The system was charge neutralized by adding Na^+^ and Cl^−^; the total ionic strength of the system was then adjusted to 0.150 M. The simulations of REJd1, -d2, -d3, and -d4 contained 9870, 8303, 13905, and 8608 atoms, respectively. Each system was then minimized to equilibrium using conjugate gradient minimization from an initial temperature of 298 K. This was followed by a 600 ps MD step to equilibrate the protein, water, and ions. For the SMD experiment, a spring constant of 10 *k*_*B*_ *T* Å^−2^ was used. Simulated force was applied by fixing the C-terminal C*α* atom in the model and pulling the N-terminal C*α* SMD atom with constant velocity along a predetermined vector. The trajectories were recorded every 2 fs and then analyzed with VMD. The REJ domains were pulled at a constant velocity of 0.001 Å·ps^−1^ and was followed for 150 Å. To validate the accuracy of our *in silico* experiments we carried out SMD simulations on titin I27. Our SMD results for I27 are very similar to those published previously [41, 43] (Figure S2).

## 3. Results and Discussion

### 3.1. Analysis of Potential FNIII Domains in the REJ Module Using Homology Modeling Techniques

Homology models for REJd1, REJd2, REJd3, and REJd4 (Figure 1(c)) were based on Clustal alignments of the primary sequences for predicted REJ domains with the primary sequences of the following template structures (Figure 1(b)): the human PKD domain no. 1 from PC1 (1b4r) and the human PKD domain from protein KIAA0319 (2e7m). The domain boundaries were based on Schröder et al. sequence analysis [24] and our Clustal multiple sequence alignment. The overall identity between each of the REJ domains and the template structures was low (~10% overall identity, ~27% similarity). Our method for homology model determination relies on finding periodicity within the primary sequence, that is, characteristic of beta-sheet structure. A similar technique was used to compute a homology model of the NS3 proteases of the Hepatitis C virus that have low sequence identity (~15%) [44]. For each homology model, 10 candidate structures were calculated. We chose the best model based on the lowest calculated model energy values as reported by MODELLER. Further, we assessed the perresidue DOPE score on the final model versus the template structure and refined any poorly scoring loop regions accordingly.

 In order to assess the overall quality of the putative REJ domains structures with respect to well-determined structures we used the programs WHAT_CHECK [45] and ERRAT2 [46]. We found that REJd1 rated the highest of the four models on the ERRAT2 scale (quality factor = 72.7). In addition, the WHAT_CHECK packing quality scored best at *Z* = −2.839, while the RMSD of REJd1 versus the template structure (1b4r) was 3.66 Å. The REJd2 model scored lower on the ERRAT2 scale (35.2), but the RMSD versus the template structure was 2.52 Å. This could be indicative of a good alignment with the template structure, but the hydrophobic core of the REJd2 domain may not provide sufficient packing within the hydrophobic core of the domain that can be measured in other FNIII type domains. REJd3 and REJd4, on the other hand, score less well by these metrics. While the REJd3 matches well with its template, 2e7m (RMSD = 2.20 Å), the packing quality score is rated as “poor” (−4.3). REJd4 scored worse than the others. The putative REJd4 domain is the most divergent of the four; it does not have a well-defined core structure. The RMSD versus its template (2e7m) was 4.02 Å, while the quality factor was very low at 23.5. This is likely because REJd4 does not have a Trp in the core region where the Trp residue seems to be conserved in the REJ folds. Hence, our homology modeling analysis shows that the primary sequences for REJd1 and REJd2 are consistent with known FNIII domains, while the REJd3 domain may represent a partially structured domain, and REJd4 most likely lack a stable tertiary structure. 

 Based on this analysis we hypothesize that the putative REJd1 and REJd2 domains may have a fold similar to the FNIII domains and that REJd3 and REJd4 most likely lacks a stable tertiary structure. In order to test this hypothesis we used SMFS and SMD methods. These methods have been successfully used by a number of groups to obtain structural information, such as the mechanical stability (Ig and FNIII domains typically unfold at much higher forces than alpha-helical domains) and the increase in contour length upon unfolding (which is proportional to the number of residues that are exposed after unfolding) [47].

### 3.2. Mechanical Signatures of REJd1 and REJd2 Domains

**Figure 2:**
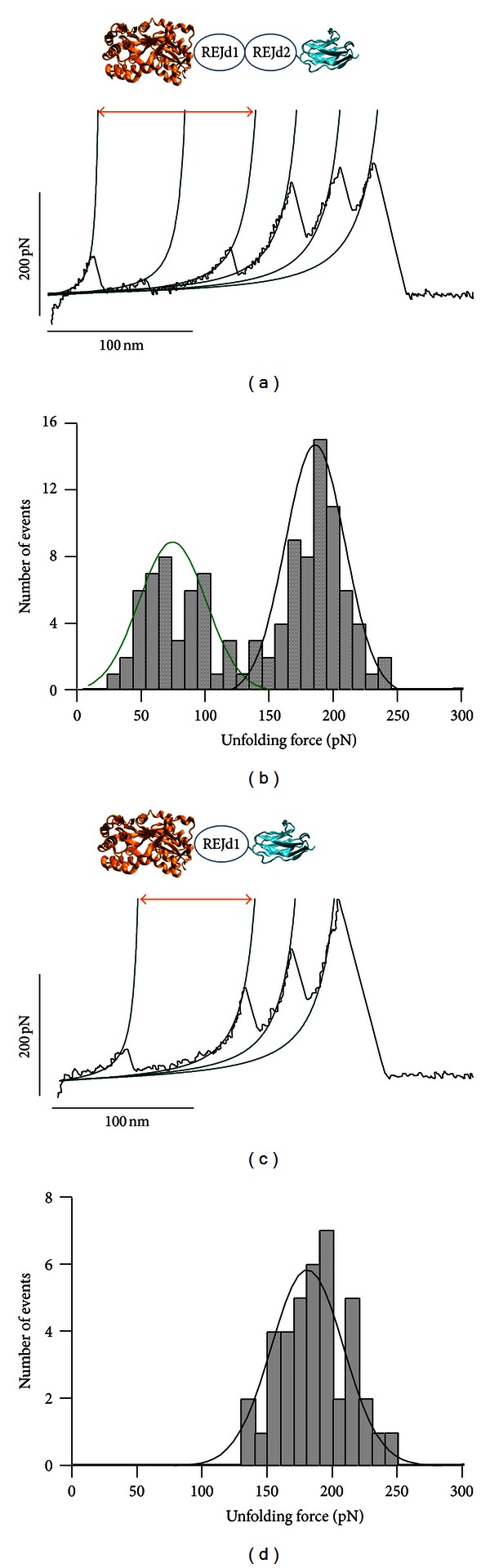
Analysis of the mechanical stability of putative REJ domains 1 and 2. (a) Typical unfolding pattern of the MBP-REJd1,2-I27 protein. The first two peaks in the force-extension curve correspond to the unfolding of MBP, with a total increase in ΔLc of about 100 nm (red double headed arrow) and an unfolding intermediate with a ΔLc of about 55 nm. We assign the third force peak to the unfolding of one of the REJ domains and the next two to the unfolding of the other REJ and I27 domains. (b) Unfolding force histogram for the I27 and REJ domains in the MBP-REJd1,2-I27 construct. In this histogram we did not include the unfolding of the MBP protein. The best fits to Gaussian distributions were obtained with the following parameters: 63 ± 37 pN (*n* = 42) and 190 ± 30 pN (*n* = 61; 47 traces). (c) Force-extension trace of the MBP-REJd1-I27 construct. This example shows the all-or-none unfolding of the MBP protein; in this example there is no unfolding intermediate. The increase in ΔLc is about 100 nm (red double headed arrow). The next two force peaks correspond to the unfolding of REJd1 and I27 domains. The black lines correspond to fits to the WLC equation using a ΔLc of 29 nm. (d) Unfolding force histogram for the I27 and REJ domains in the MBP-REJd1-I27 construct. In this histogram we did not include the unfolding of the MBP protein. There is a single distribution of force peaks with a mean of about 190 pN (188 ± 39 pN, *n* = 40; 21 traces).

 We found that the MBP-REJd1-I27 and MBP-REJd1,2-I27 constructs are expressed as soluble proteins in *E. coli*. The advantage of using MBP and I27 proteins is that they provide unique mechanical unfolding fingerprints. Both MBP and I27 have been characterized using SMFS techniques [30, 48–51]. Stretching the construct containing MBP, titin I27 domain and sequences for REJd1,2 generated sawtooth patterns with distinctive force peaks and increases in contour lengths, ΔLc (Figure 2(a)). To determine the contribution of each domain to the unfolding pattern we analyzed the spacing between peaks in the unfolding patterns. We used the worm-like chain (WLC) model for polymer elasticity, which predicts the entropic restoring force generated upon the extension of a polymer [38, 39]. The thin lines in Figure 2(a) correspond to manual fits of the WLC equation to the curve that precedes each force peak. The I27 domains have been shown to unfold at forces of ~200 pN and produce an increase in contour length (ΔLc) of ~29 ± 8 nm upon unfolding [30]. On the other hand, MBP is known to unfold at forces of about 70 pN with a total increase in ΔLc of ~100 nm upon unfolding [49, 50]; it was found that MBP can also unfold via a mechanically stable unfolding intermediate which contributes a ΔLc of ~50 nm upon unfolding [50]. Hence, we attribute the first 100 nm of the recording to the unfolding of the MBP protein. This trace also shows the unfolding intermediate. There are three force peaks before the detachment from the surface; these all show a ΔLc of about 29 nm. One of them has a peak force of about 70 pN. Given the construction of the protein this means that the REJd1 and REJd2 unfold at very different forces, one at about 70 pN and the other a force similar to the I27 domain (~200 pN).  Figure 2(b) shows an unfolding force histogram for the REJ and I27 domains. There are two clear populations, one unfolds at a low force of 63 ± 37 pN (*n* = 42) and the other at 190 ± 30 pN (*n* = 61).

 Although we can confidently discriminate between one of the REJ domains and the I27 titin domain with these data, the identity of the individual REJ domains in these recordings cannot be established at this point. To unambiguously identify the force peaks from each REJ domain, we constructed a protein containing REJd1 with a flanking MBP and an I27 domain.  Figure 2(c) shows a trace obtained after stretching the MBP-REJd1-I27 protein. This example shows the MBP protein unfolds in an all-or-none unfolding manner with no unfolding intermediate. The increase in ΔLc is about 100 nm. The next two peaks have unfolding forces of 148 pN and 204 pN, respectively. One of these events must correspond to the unfolding of the REJd1 domain. However, a precise assignment cannot be made since the unfolding force histogram shows only one distribution centered at ~190 pN (188 ± 39 pN, *n* = 40; Figure 2(d)). 

Based on these data we can conclude that REJd1 unfolds at similar forces rather than the I27 domain (~200 pN), and REJd2 unfolds at a significantly lower force (~60 pN).

### 3.3. Mechanical Signatures of REJd3 and REJd4 Domains

**Figure 3:**
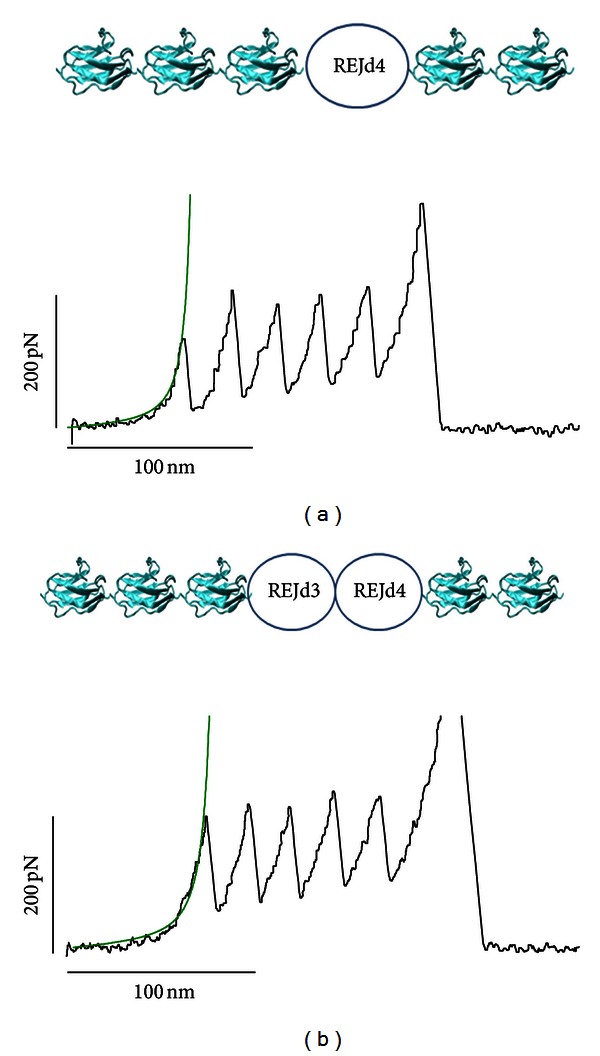
Analysis of the mechanical stability of putative REJ domains 3 and 4. (a) Example of unfolding pattern observed after stretching the (I27)_3_-REJd4-(I27)_2_ protein. (b) Example of unfolding pattern observed after stretching the (I27)_3_-REJd3,4-(I27)_2_ protein. In these two examples the unfolding of the five titin I27 domains (they unfold on average at 200 pN with an increase length of 28 nm) is preceded by a long spacer.

 In order to study the mechanical unfolding of putative REJ domains REJd3 and REJd4 we constructed a protein containing the REJd3 and REJd4 sequences plus MBP and I27 (MBP-REJd3,4-I27). However, we found that this is construct expressed poorly and mostly in inclusion bodies in *E. coli*. To further increase the solubility we used a chimeric I27 polyprotein approach that has been successfully used to study proteins that tend to aggregate such as alpha-synuclein, huntingtin polyQ, and tau proteins [52–54]. For this purpose we inserted the REJd3,4 sequence in between several I27 domains. We made two constructs in this way, (I27)_3_-REJd4-(I27)_2_ and (I27)_3_-REJd3,4-(I27)_2_. These are expressed well as soluble proteins in *E. coli*.  Figure 3 shows examples obtained after stretching these constructs. To facilitate the analysis we selected traces that had five I27 unfolding peaks and had a clean initial force extension after retraction from the surface (i.e., little or no unspecific interactions). In the case of the (I27)_3_-REJd4-(I27)_2_ and (I27)_3_-REJd3,4-(I27)_2_. These recordings show only five unfolding peaks. There are two possible scenarios that the mechanical stabilities of REJd3 and REJd4 are much higher or lower (within the noise) than those for the I27 domain. It is unlikely that the mechanical stabilities of REJd3 and REJd4 exceed that of titin I27 because: (i) we observed no more than five force peaks that show the mechanical fingerprint of I27 domains (i.e., unfolding at ~200 pN and an interpeak spacing of ~28 nm), (ii) the detachment forces (last force peak) are >400 pN and all protein domains studied so far unfold at forces less than this force [37], and (iii) we typically observed a spacer before the unfolding of the I27 domains. In the example shown in Figure 3(a), the distance to the first I27 force peak is about 60 nm, and in Figure 3(b) this distance is about 85 nm. The spacers observed in Figure 3 are characteristically seen in domains that have a mostly disordered or random coil conformation, such as, for example, tropoelastin, the titin PEVK domain, or some neurotoxic proteins [52–55]. In addition Far-UV CD analysis is also consistent with REJd4 forming an unstructured random coil (Figure S1). The spectra for the (I27)_3_-REJd4-(I27)_2_ protein shows a significant higher random-coil content (43%) than that of a (I27)_8_ protein (35%).

 Hence, we conclude that domains REJd3 and REJd4 form mechanically weak structures (random-coil or unfolded conformation) that unfold at forces that are below the resolution of our AFM (<10 pN). 

### 3.4. Steered Molecular Dynamic Simulations of REJ FNIII Domains

**Figure 4:**
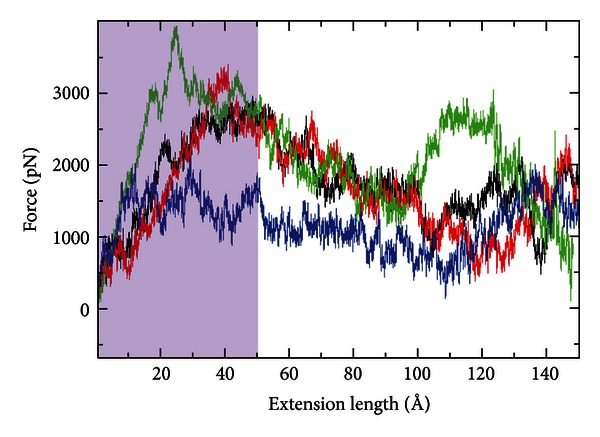
Constant velocity steered molecular dynamics simulations of the mechanical unfolding of REJ domains. Constant velocity steered molecular dynamics simulation of the mechanical unfolding of REJd1 (black), REJd2 (red), REJd3 (green), and REJd4 (blue). Force-extension curves were obtained from the SMD simulation of each REJ domain model by first fixing the C-terminal C*α* atom and then applying a constant force to the N-terminal C*α* atom along a predetermined vector. Forces (in pN) were recorded for each time step along the simulation. The shaded area corresponds to the initial burst of force that is typical of other FNIII domains.

 As our homology models of REJd1-d4 are based on preexisting structures, we sought to assay the biophysical correspondence between our *in silico* domains and experimental data. Steered molecular dynamic simulations (SMDs) analysis has been used in the past to study the mechanical unfolding pathways of FNIII domains [56–59]. For example the simulated force-extension curves for FNIII domain no. 10 of fibronectin show a single dominant force peak which corresponds to the rupture of the tertiary structure of FNIII [59].  Figure 4 shows constant velocity SMD simulations of the mechanical unfolding of the four REJ domains. The force-extension curves were obtained from SMD simulations by stretching domains between its C-terminus and its N-terminus at a pulling speed of 0.001 Å·ps^−1^. The magnitude of the forces observed in the SMD simulations does not directly correspond to those measured with AFM. This is partially because the pulling speeds are several orders of different magnitude. However, the simulations are qualitatively consistent with AFM experiments [60–62]. To validate the accuracy of our *in silico* experiments we carried out SMD simulations on titin I27. Our SMD results for I27 are very similar to those published previously [41, 43] (Figure S2). Our simulations show that force-extension profiles of REJd1 and REJd2 are very similar and show a force peak of about 3000 pN at around 40 Å. The shaded area corresponds to the initial burst of force that is typical of other FNIII domains [56–59]. 

 Both the REJd1 (black curve) and REJd2 (red curve) agree with experimental measurements of the REJd1 and REJd2 proteins. Further, the blue SMD force-curve (REJd4) agrees with our experimental data for REJd4; that is, it is likely not well folded. However, there are clearly limitations to the current computational techniques. While the primary sequence that we have assigned as REJd3 fits to an Ig-like model and it reacts like a properly folded FNIII-like domain in our SMD simulation, it is clearly intermediate between a folded and a nonfolded domain for reasons that SMD technique is not accurate enough to simulate.

## 4. Conclusions

 The available evidence indicates that PC1 has a role in sensing of flow [2, 3], pressure [4], cell cycle [5], cell polarity regulation [6], and kidney development [63]. PC1 may sense signals from the primary cilia, neighboring cells, and extracellular matrix and transduces them into cellular responses that regulate proliferation, adhesion, and differentiation that are essential for the control of renal tubules and kidney morphogenesis [1, 3, 7, 8].

 In this work we combined homology modeling, protein engineering SMD simulations, and SMFS to systematically characterize the mechanical stability of the predicted four FNIII domains in the REJ module of PC1. Of the 80 missense mutations in the REJ module about 20 disease-causing mutations map onto the region studied in this work. After flanking the different putative FNIII sequences with titin I27 or MBP domains we found that these constructs were expressed well as soluble proteins in *E. coli* and were able to analyze their mechanical stability. Our study provides direct mechanical force measurements of four putative FNIII domains in the REJ module and provides *in vitro* evidence that these domains have a different mechanical stability. Stretching a construct containing the REJd1 and REJd2 sequences generated mechanical fingerprints that correspond to the unfolding of the MBP and I27 domains as well as extra unfolding events that represent the unfolding of the REJd1 and -d2 domains. Our data show that REJd1 and REJd2 domains are mechanically stable and unfold at forces of about 200 pN and 60 pN, respectively. This range of values is consistent with those reported for the mechanical unfolding of FNIII domains [64, 65]. We found that constructs harboring the REJd3 and REJd4 sequences are expressed well as soluble proteins in *E. coli *or insect cells (Sf9); however they do not form mechanically stable folded domains. Our results do not exclude the possibility that REJd3 and REJd4 form stable folds when expressed in other cells, such as human kidney epithelial cells, or when expressed within the native full length extracellular region. It is also possible that these domains need molecular chaperones to acquire a proper stable fold. Hence, future experiments in more physiological settings would be necessary to resolve this important issue and facilitate the univocal characterization of these domains. The REJ module of PC1 could represent a continuation of the PKD domain structure known to exist in PC1. That is, the protein could possess a series of PKD domains that terminate in a series of homologous but mechanically weaker FNIII domains. It is possible that the primary sequence of the REJd3 and REJd4 repeating units degraded over evolution into structures that still retained the general character of the parental FNIII domain but do not possess the mechanical stability preset in the more N-terminal domains. We speculate that the REJd3 and REJd4 domains may function as entropic springs designed to adjust the length of the extracellular region of PC1 in response to mechanical shear stress. This spring-like behavior might also be important for the autoproteolysis in the GPS domain, a process that requires the complete REJ module. 

 We and others (e.g., [13, 66]) have shown that SMFS techniques can be used to accurately quantify the effects of disease causing mutations on single protein domains. Pathogenic missense mutations that target PC1 PKD domains were shown to result in a loss in mechanical stability which may lead to the abnormal mechanical function of PC1 [13]. Our SMFS results demonstrate a powerful experimental approach to study the domain architecture and stability of the REJ module and should pave the way to systematically characterize the effects of disease-causing mutations in the REJ module of human PC1.

## Supplementary Material

The Supplementary Material contains far-UV CD spectroscopy of the (I27)3-REJd4-(I27)2 protein construct and a control plot of a steered molecular dynamics simulations of the mechanical unfolding of titin domain I27.

## Figures and Tables

**Table 1:** REJ constructs used for SMFS experiments.

Protein construct	Amino acid (human PC1) Genbank no. L33243	Expression system	Remarks	Expression vector
REJd1	2151–2256	Insect cell Sf9	Very low expression/insoluble	pFastBac
REJd4	2468–2575	Insect cell Sf9	Very low expression/insoluble	pFastBac
REJd1-4	2151–2575	Insect cell Sf9	Very low expression/insoluble	pVL1392
MBP-REJd1-I27	2151–2256	*E. coli *	Good expression/soluble	p202
MBP-REJd1,2-I27	2151–2375	*E. coli *	Good expression/soluble	p202
MBP-REJd3,4-I27	2380–2575	*E. coli *	Very low expression/insoluble	p202
(I27)_3_-REJd3,4-(I27)_2_	2380–2575	*E. coli*/Sf9	Good expression/soluble	pRSETA/pVL1392
(I27)_3_-REJd4-(I27)_2_	2468–2575	*E. coli*/Sf9	Good expression/soluble	pRSETA/pVL1392

MBP: maltose-binding protein; I27: titin domain I27.

## Abbreviations

AFM: Atomic force microscopy
SMFS: Single-molecule force spectroscopy
SMD: Steered molecular dynamics
REJ: Receptor for egg jelly
PC1: Polycystin-1.
