# Supplementary material for: Analysis of the REJ Module of Polycystin-1 Using Molecular Modeling and Force-Spectroscopy Techniques
Source: J Biophys. 2013 May 26;2013:525231. doi: 10.1155/2013/525231 (PMC3677617; doi:10.1155/2013/525231)
Supplement: Supplementary file 1 — The Supplementary Material contains far-UV CD spectroscopy of the (I27)3-REJd4-(I27)2 protein construct and a control plot of a steered molecular dynamics simulations of the mechanical unfolding of titin domain I27. [file 525231.f1.doc]

**Supplemental Material**

“Analysis of the REJ module of Polycystin-1 using Molecular Modeling and Force-Spectroscopy Techniques”

Meixiang Xu, Liang Ma, Paul Bujalowski, Feng Qian, R. Bryan Sutton and Andres F. Oberhauser


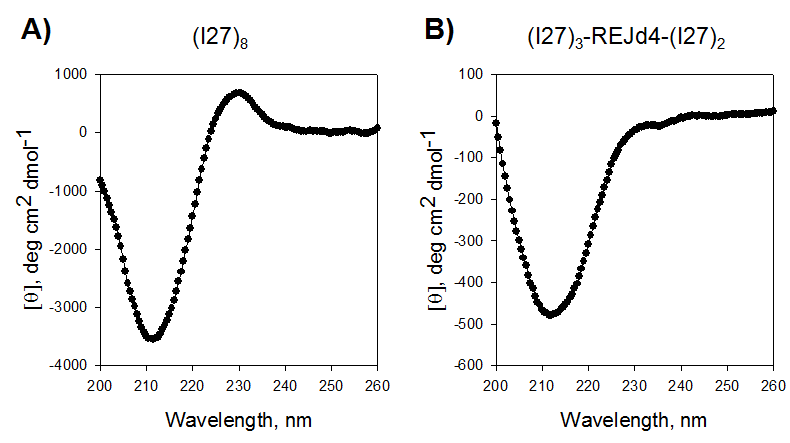


**Figure S1. Far-UV CD spectroscopy of the REJd,4-I27 protein chimera.** CD spectra for a titin I27 polyprotein (A) and the (I27)_3_-REJd4-(I27)_2_ protein (B). Both show a minimum at 211 nm, however the beta-structure and random-coil content, as estimated by the CDNN program was significantly different. The alpha-helix, beta-structure and random coil were 17%, 48%, 35% and 13%, 44%, 43% for the I27 and REJd4 constructs, respectively.

**Figure S2. Constant velocity steered molecular dynamics simulations of the mechanical unfolding of titin domain I27.** The force-extension curve was obtained from the SMD simulation by first fixing the C-terminal Cα atom (Leu1) and then applying a constant force to the N-terminal Cα atom (Leu 89) along a pre-determined vector. Forces (in pN) were recorded for each timestep along the simulation.
